# Simultaneous bioethanol distillery wastewater treatment and xylanase production by the phyllosphere yeast *Pseudozyma antarctica* GB-4(0)

**DOI:** 10.1186/s13568-015-0121-8

**Published:** 2015-06-12

**Authors:** Takashi Watanabe, Ken Suzuki, Ikuo Sato, Tomotake Morita, Hideaki Koike, Yukiko Shinozaki, Hirokazu Ueda, Motoo Koitabashi, Hiroko K Kitamoto

**Affiliations:** National Institute for Agro-Environmental Sciences (NIAES), 3-1-3 Kannondai, Tsukuba, Ibaraki 305-8604 Japan; Japan Society for the Promotion of Science, 1-8 Chiyoda-ku, Tokyo, 102-8472 Japan; Research Institute for Innovation in Sustainable Chemistry, National Institute of Advanced Industrial Science and Technology (AIST), Tsukuba Central 5-2, 1-1-1 Higashi, Tsukuba, Ibaraki 305-8565 Japan; Bioprocess Research Institute, National Institute of Advanced Industrial Science and Technology (AIST), Tsukuba Central 6, 1-1-1 Higashi, Tsukuba, Ibaraki 305-8566 Japan

**Keywords:** Xylanase, *Pseudozyma antarctica*, Jar-fermentor, Xylose inducible, Lignocellulosic bioethanol distillery wastewater, Wastewater treatment

## Abstract

Bioethanol production using lignocellulosic biomass generates lignocellulosic bioethanol distillery wastewater (LBDW) that contains a large amount of xylose, making it a potential inexpensive source of xylose for biomaterials production. The main goal of this study was the production of useful enzymes from LBDW during treatment of this wastewater. In this study, we found that xylose strongly induced two yeast strains, *Pseudozyma antarctica* T-34 and GB-4(0), to produce novel xylanases, PaXynT and PaXynG, respectively. The nucleotide sequence of PaXynT [accession No. DF196774 (GAC73192.1)], obtained from the genome database of strain T-34 using its N-terminal amino acid sequence, was 91% identical to that of PaXynG (accession No. AB901085), and the deduced amino acid sequence is 98% identical. The specific activities of the purified PaXynT and PaXynG were about 52 U/mg. The optimal pH and temperature for both enzymes’ activities were 5.2 and 50°C, respectively. They hydrolyzed xylan to xylose and neither had *β*-xylosidase (EC 3.2.1.37) activity, indicating that they are endo-*β*-xylanases (EC 3.2.1.8). With these results, we expect that PaXyns can be employed in saccharizing lignocellulosic biomass materials for the production of useful products just like other endoxylanases. After 72 h of LBDW fed-batch cultivation using a jar-fermentor, strain GB-4(0) produced 17.3 U/ml (corresponding to about 0.3 g/l) of PaXynG and removed 63% of dissolved organic carbon and 87% of dissolved total phosphorus from LBDW. These results demonstrate the potential of *P. antarctica* for xylanase production during LBDW treatment.

## Introduction

Wastewater treatment system that uses yeasts could remove large amounts of organic compounds (10,000 COD mg/l/day), requires little space, and discharges little waste sludge (Yoshizawa 1978). This method is useful for treating food, brewing, and beverage industry wastewater (Yoshizawa 1978; Watanabe et al. 2009). Furthermore, the utilization of high strength wastewater is considered as an attractive way to produce useful materials, single cell protein, lactic acid, methane, and enzymes (lipase and protease) (Siso 1996; Angenent et al. 2004). Accordingly, using yeasts were suitable for high yield production of useful materials from high strength wastewater (Watanabe et al. 2013a, 2014a).

Producing bioethanol from lignocellulosic biomass generates lignocellulosic bioethanol distillery wastewater (LBDW) that contains high concentrations of organic compounds (30,000–60,000 COD mg/l). The cost of LBDW treatment directly increases the cost of bioethanol production. Since the ethanol-producing yeast *Saccharomyces cerevisiae* does not ferment xylose (Matsushika et al. 2008), the resulting LBDW is found to contain a large amount of xylose, making it a potential inexpensive source of fermentable xylose.

A non-pathogenic basidiomycetous yeast, *Pseudozyma antarctica* has an ability to assimilate various kinds of carbon sources (Boekhout 2011). Recently, we found that *P.antarctica* GB-4(0) efficiently removed organic compounds from *shochu* (a Japanese traditional distilled liquor) distillery wastewater (Watanabe et al. 2013b). The whole genome sequence of *P.antarctica* T-34 had already been determined and annotated (Morita et al. 2013). Moreover, we discovered that *P. antarctica* produces a cutinase-like enzyme (CLE), designated as PaE (22-kDa), which efficiently degrades various synthetic biodegradable polyesters (Kitamoto et al. 2011; Shinozaki et al. 2013), and that the PaE productivity was increased by xylose (Watanabe et al. 2014b). For scale-up production of PaE, under xylose feeding cultivation of *P. antarctica* using a jar fermentor (Watanabe et al. 2014b), we found a highly secreted 33-kDa unknown protein (unpublished data). If this 33-kDa unknown protein is a useful enzyme, it could be efficiently produced from large amounts of cheap xylose containing in LBDW during treatment.

A basidiomycetous yeast, *Cryptococcus* sp. strain S-2 (Masaki et al. 2012) and a yeast-like fungus, *Aureobasidium pullulans* (Dobberstein and Emeis 1989) secrete xylanases from xylose. Endo-*β*-xylanases (EC 3.2.1.8), which hydrolyze xylopyranosyl linkages of *β*-1,4-xylan, the principal type of hemicellulose, are useful for saccharizing lignocellulosic biomass materials for the production of bioethanol and other useful chemicals (Biely 1985). Xylanases are also utilized in paper and pulp industries (Pokhrel and Viraraghavan 2004) and pretreatment of animal feeds (Beg et al. 2001). The utilization of LBDW, a cheap substrate for xylanase production, would be an attractive way to reduce xylanase production costs, while treating LBDW.

In this study, we attempted to purify and identify the 33-kDa unknown proteins from *P. antarctica* T-34 and GB-4(0) using the genomic DNA sequence of T-34. After confirming that these proteins were xylanases, their biochemical properties were characterized. The xylanase of GB-4(0) was produced from LBDW derived from rice straw hydrolysate in large scale using a jar-fermentor.

## Materials and methods

### Strains

*Pseudozyma antarctica* GB-4(0) was isolated from rice husks (Kitamoto et al. 2011) whereas *P.antarctica* T-34 was isolated from Mt. Tsukuba soil sample (Kitamoto et al. 1990). These strains were deposited in the Genebank at the National Institute of Agrobiological Sciences (NIAS), Japan [accession numbers: T-34, MAFF 306900; GB-4(0), MAFF 306999].

### Cultivation conditions

The two yeast strains were pre-cultivated in test tubes containing 5 ml of YM medium (0.3% yeast extract, 0.3% malt extract, 0.5% peptone, 1% dextrose) at 30°C with reciprocal shaking at 160 rpm for 24 h. The pre-cultures (100 μl) were added to 100 ml flasks containing 20 ml of modified fungal minimum medium (FMM) (0.3% yeast extract, 0.2% NaNO_3_, 0.06% KH_2_PO_4_, 0.06% MgSO_4_·7H_2_O) with 8% xylose or beechwood xylan. Xylose and other nutrients were separately autoclaved (121°C, 20 min). The cultures were cultivated at 30°C with rotary shaking at 200 rpm for 96 h.

Every 24 h, 1-ml aliquots of the culture were harvested and centrifuged at 15,000 rpm for 5 min. The pellets were dried at 105°C for 2 h and their dry cell weights were measured to investigate cell growth. At the same time, the xylanase activities of the supernatant were measured as described below.

### Enzyme activity

Xylanase activity was determined using xylan as the substrate at 30°C for 30 min. The reaction mixture contained 15 mM sodium acetate buffer (pH 5.2), 1.0% (w/v) beechwood xylan (Sigma-Aldrich Japan), and 100 μl of culture supernatant in a total volume of 1 ml. After the reaction, the amount of reducing sugar was analyzed by the modified Somogyi–Nelson method (Hatanaka and Kobara 1980) with d-xylose as the standard. One unit (U) of xylanase activity was defined as 1 μmol of d-xylose liberated per min in the reaction mixture.

*β*-Xylosidase activity was also determined using the synthetic substrate *p*-nitrophenyl-β-d-xylopyranoside (pNP-X) (Iefuji et al. 1996). The reaction mixture contained 200 μl of 4 mM pNP-X solution, 600 μl of 30 mM sodium acetate buffer (pH 5.2) and 200 μl of enzyme solution in a total volume of 1 ml. After 30 min, 2 ml of 0.2 M Tris–HCl buffer (pH 8.5) was added to stop the enzyme reaction. One unit of *β*-xylosidase activity was defined as 1 μmol of *p*-nitrophenol released per min.

### Electrophoresis

Sodium dodecyl sulfate-polyacrylamide gel electrophoresis (SDS-PAGE) was performed according to the method of Laemmli (1970) using a 14.1% polyacrylamide slab gel. Proteins were visualized by Coomassie brilliant blue (CBB) staining using Phast Gel Blue R (GE Healthcare) and by silver staining (2D-Silver stain II “Daiichi”; Daiichi Pure Chemicals Co., Ltd. Tokyo, Japan).

The proteins were transferred to PVDF membranes (Pall, Port Washington, NY, USA) by semidry blotting. The 33-kDa band corresponding to *P.antarctica* T-34 protein, which was designated as PaXynT was excised and its N-terminal amino acid sequence was determined using ABI Procise 491HT (Applied Biosystems, Foster City, CA, USA).

### Genomic DNA isolation

The two yeast strains were cultivated in 5 ml of YM medium overnight. The cultures were centrifuged, and genomic DNA was isolated from the cell pellets using Dr. GenTLE for Yeast (Takara, Kyoto, Japan).

### Sequencing of the PaXyn genes

The N-terminal peptide sequence of the PaXynT was searched in the annotated whole genome sequences of *P. antarctica* T-34 (Morita et al. 2013), and was used to obtain its genomic sequence. To determine the nucleotide sequence of the unknown protein produced by *P. antarctica* GB-4(0), designated as PaXynG, forward primers (PaXynF1, PaXynF2, PaXynF3, and PaXynF4) and reverse primers (PaXynR1, PaXynR2, PaXynR3, and PaXynR4) were designed based on the genomic sequence of PaXynT. The primer sequences and their positions in the T-34 gene are shown in Table 1. The entire gene for PaXynG was obtained by PCR amplification using the above primers and genomic DNA of *P. antarctica* GB-4(0) as template. The reaction was performed using TaqEX (Takara, Kyoto, Japan) according to the manufacturer’s instructions. Amplified DNA fragments were subjected to 1% agarose gel electrophoresis and were purified using QIAquick Gel Extraction Kit (Qiagen, Hilden, Germany). The sequences of the PCR products were determined using BigDye Terminator Cycle Sequence V3.1 Kit with the same primers and Applied Biosystems 3100 Genetic Analyzer (Applied Biosystems, Foster City, CA, USA).

**Table 1:** Primers used for sequencing of PaXynG gene of *P. antarctica* GB-4(0)

Primer name	Primer sequence	Position^a^
PaxynF1	5′-GAAGGCTGAAGCTTTGGCTCTGACAT-3′	−600 to −574
PaxynF2	5′-CATGCTTGAAGCTCCAAGAAGATATAA-3′	−120 to −93
PaxynF3	5′-CACTCGCAGCTGCCTTCGTGGGTGCAG-3′	361 to 387
PaxynF4	5′-GAAGGCAGTCTGCTCGGCCGCTCCCGA-3′	841 to 867
PaxynR1	5′-TGTGGTGTTTGTTTGGCGTTTTTGCTT-3′	0 to −26
PaxynR2	5′-ATCCCACGCGTACACCTTGCCCTTGTA-3′	480 to 453
PaxynR3	5′-CCCACGCAGTTGGACTGGGCCAGGCAG-3′	960 to 933
PaxynR4	5′-CGAGCGCGATTTTCTCCGAGTCTAAA-3′	1,390 to 1,365

^a^Positions of the nucleotides in the PaXynT gene sequence of *P. antarctica* T-34.

### Purification

After 96 h flask cultivation of both strains, the culture supernatants obtained after centrifugation (7,000×*g*, 10 min, 4°C) were filtered using a membrane filter (ADVANTEC^®^ C045A090C, 0.45 μm, Toyo Roshi Kaisha, Ltd. Japan). One-ml aliquots of filtrates were applied to a TSK-GEL3000SW_XL_ column (Tosoh) in 50 mM acetate sodium buffer (pH 5.2) containing 0.3 M NaCl at a flow rate of 0.5 ml/min. The absorbance was measured at 280 nm. Fractions containing enzyme activity were concentrated, desalted using Amicon Ultra-15 10000 MWCO (MILLIPORE), and filtered using a syringe filter (ADVANTEC^®^ DISMIC^®^-25cs, 0.2 μm, Toyo Roshi Kaisha, Ltd.). The filtrate (100 μl) was mixed with 900 μl of 50 mM Na-phosphate buffer (pH 7.1) containing 1.3 M ammonium sulfate. The mixture (1 ml) was applied to a hydrophobic interaction column (Phenyl Superose HR 5/5; GE Healthcare UK Ltd.) with a linear gradient (1.2–0 M) of ammonium sulfate in 50 mM Na-phosphate buffer (pH 7.1) at a flow rate of 0.5 ml/min. Fractions containing enzyme activity were concentrated, desalted, and filtered as described above. Protein concentration was measured with a protein assay kit (Bio-Rad Laboratories, Hercules, CA, USA).

### Enzyme pH and temperature properties

Optimal pH for enzyme activity was determined at 30°C for 30 min with beechwood xylan as substrate using several 15 mM buffers: sodium acetate-HCl (pH 2.0–4.0), sodium acetate (pH 4.0–5.2), sodium phosphate (pH 5.2–6.8), and Tris–HCl (pH 6.8–8.0). To estimate pH stability, the enzyme solutions were incubated at pH ranging from 2.0 to 8.0 using the above buffers for 1 h at 30°C. The residual xylanase activity was then determined at pH 5.2. Optimal temperature for activity was determined at pH 5.2 for 30 min at different temperatures (30–70°C) with beechwood xylan as substrate. Thermostability was estimated by incubating the enzyme sample in water bath at 30–70°C for 30 min. After heat treatment, samples were chilled on ice and residual xylanase activity was determined as described above.

### Xylan hydrolysis and end product analysis

Purified PaXynG was used to determine the end products of beechwood xylan hydrolysis. The reactions were conducted at pH 5.2 and 30°C. After incubations at 10, 20, and 30 min, the reactions were stopped by boiling. The hydrolysis products were separated by thin-layer chromatography (TLC) according to the method of La Grange et al. (2001). Samples of the reaction mixtures, containing 20 μg of xylose-equivalent sugars, were applied to a TLC sheet (Silica gel 60 F_254_, Merck, Darmstadt, Germany) that was placed in a chamber containing 7:1:2 solvent mixture of *n*-propanol:ethanol:H_2_O. A xylooligosaccharides standard mixture (20 μg/μl) containing xylose (X1), xylobiose (X2), xylotriose (X3), and xylotetraose (X4) was also used. The sugar spots were identified by spraying 1% anthrone dissolved in 75% sulfuric acid, followed by heating (Morita et al. 2010). Hydrolyzed xylose was measured using a d-xylose assay kit (Megazyme, Ireland) according to the manufacturer’s manual.

### Jar-fermentor cultivation

*Pseudozyma antarctica* GB-4(0) was used for the jar-fermentor cultivation experiments. A 30-ml pre-culture was grown in a 300-ml flask at 30°C with rotary shaking at 200 rpm for 24 h in YM medium. This culture was then used to inoculate the 5-l jar fermentor containing 3 l of PaXyn production medium [0.2% yeast extract, 0.2% NaNO_3_, 0.2% (NH_4_)_2_SO_4_, 0.04% KH_2_PO_4_, 0.04% MgSO_4_·7H_2_O, and 2% xylose]. Batch cultivation was performed until all the xylose was depleted (around 24 h). Then, xylose-fed-batch cultivation was performed to induce PaXynG production by adding fed medium [0.2% yeast extract, 0.085% YNB w/o amino acid and ammonium sulfate (Difco) and 20% xylose] at a feeding rate of 500 ml/day using a peristaltic pump. Xylose and other nutrients were separately autoclaved (121°C, 20 min).

LBDW derived from rice straw hydrolysate was kindly provided by Biomaterial in Tokyo Co., Ltd. The concentration of xylose (6.7%) was measured with a d-xylose assay kit (Megazyme, Ireland). Analysis of LBDW using standard methods (APHA et al. 1998) showed the following components: dissolved organic carbon (DOC), 52,000 mg/l, dissolved total nitrogen (DTN), 550 mg/l, and dissolved total phosphorus (DTP), 600 mg/l. It has a pH of 4.2. The pre-culture (30 ml) prepared as described above was inoculated into 2–l of 4-times-diluted LBDW. After 24 h cultivation, LBDW was continuously added at a rate of 1 l/day using a peristaltic pump.

The cultivation conditions were as follows: aeration rate was 2 LPM; agitation value was 500 rpm; dissolved oxygen (DO) was maintained at around 25% of saturated value; pH was controlled at 6.0 with 14% ammonia solution, which also provided a nitrogen source for the culture; temperature was 30°C.

### Accession number

The accession number of the genomic sequence encoding PaXynG of *P. antarctica* GB-4(0) registered in DBBJ is AB901085.

## Results

### Production of xylanase from *P. antarctica* GB-4(0)

**Figure 1:**
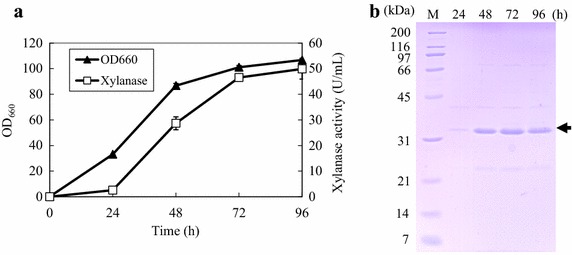
Time course of xylanase (PaXynG) production by *P. antarctica* GB-4(0) with modified FMM containing 8% xylose in flask cultivation. **a** Cell growth (*closed triangles*) and xylanase (*open squares*) production. Each result is the average of three different experiments. *Error bars* show standard deviations. **b** SDS-PAGE of supernatants (5 μl) periodically sampled from the flask cultivation. The *arrow* at 33 kDa indicates PaXyn bands.

When cultivated in flask with modified FMM with 8% xylose, *P antarctica* GB-4(0) produces a 33-kDa unknown protein (Figure 1b, indicated by the arrow). Productions of some xylanases are reported to have been induced by xylose (Furukawa et al. 2009; Gielkens et al. 1999; Masaki et al. 2012; Dobberstein and Emeis 1989). Among the *Pseudozyma* species found to have the ability to secrete xylanases are *P. hubiensis* NCIM3574 (Adsul et al. 2009) and *P. brasiliensis* sp. nov. strain GHG001 (Borges et al. 2014). These findings led us to speculate that the 33-kDa unknown protein produced by *P.antarctica* strains could also be xylanases. With this assumption, we investigated the time course of xylanase activity of *P. antarctica* GB-4(0) culture.

Xylanase activity was detected in the culture and was found to have gradually increased with time (Figure 1a). At 96 h, 49.9 U/ml of xylanase activity and cell growth with an OD_660_ of 106.7 were obtained (Figure 1a). Simultaneously, the intensity of the 33-kDa band of GB-4(0) on the SDS-PAGE gel increased with time (Figure 1b). Xylan, being the substrate of xylanases, generally induces xylanase production by microorganisms. However, with modified FMM using 8% xylan instead of xylose under the same cultivation conditions described previously, *P.antarctica* GB-4(0) showed less xylanase activity (14.0 U/ml). The 33-kDa band was also detected in this culture by SDS-PAGE analysis (data not shown).

### Nucleic acid and amino acid analysis of the two xylanases

Another *P. antarctica* strain, T-34, also produced xylanase (18.5 U/ml) under the same conditions with xylose, and the 33-kDa unknown protein band was also detected on SDS-PAGE gel (data not shown). In order to identify the xylanase of strain GB-4(0), we referred to the DNA sequence of the gene that encodes the 33-kDa unknown protein of strain T-34 based on the database for its whole genome sequence (Morita et al. 2013).

The N-terminal sequence (amino acids 35–44 after cleavage of a signal peptide) of the 33-kDa unknown protein from *P. antarctica* T-34 indicated that it corresponded to a gene with accession No. DF196774 (GAC73192.1) in the genome of *P. antarctica* T-34. This gene contains the putative promoter region (600 bp), 1,026-bp ORF, the putative terminator region (300 bp), and one putative intron (624–688) comprised of 64 nucleotides.

The deduced amino acid sequence contains 341 amino acid residues (accession No. M9ME65) and the predicted size of the mature protein (307 amino acid residues, 32.9 kDa) is almost the same as that estimated by SDS-PAGE electrophoresis. A search of DDBJ (http://www.ddbj.nig.ac.jp/) yielded a number of xylanases belonging to glycosyl hydrolases (GH) 10 family, whose amino acid sequences are 57–77% identical to the 33-kDa unknown protein of *P. antarctica* T-34 (Table 2). These results lend support to our speculation that the 33-kDa unknown protein is also a xylanase (PaXyn).

**Table 2:** Percentages of sequence identity between PaXynT of *P. antarctica* T-34 and other xylanases

Microorganism	Putative function	Accession no.	Identity (%)	Protein size (aa)
*P. antarctica* T-34	Endo-1,4-β-d-xylanase	M9ME65	–	341
*Ustilago hordei*	Probable endo-1,4-β-xylanase	I2FWP8	77	342
*Sporisorium reilianum*	Probable endo-1,4-β-xylanase	E7A3D3	75	343
*Aspergillus oryzae*	Endo-1,4-β-xylanase F3	Q96VB6	65	323
*Aspergillus sojae*	Endo-1,4-β-d-xylanase	Q9P955	64	323
*Penicillium oxalicum*	Endo-1,4-β-xylanase	E1B2N4	61	330
*Paecilomyces aerugineus*	Endo-β-1,4-xylanase	G8ZAH1	61	326
*Penicillium citrinum*	Endo-1,4-β-xylanase	B1B533	60	327
*Aspergillus terreus*	Probable endo-1,4-β-xylanase C	Q0CBM8	59	326
*Thermoascus aurantiacus*	Endo-1,4-β-xylanase	P23360	57	329

The genomic sequence encoding PaXynT of *P. antarctica* T-34, was used to design primers for the amplification of the genomic sequence encoding PaXynG of *P. antarctica* GB-4(0). A single PCR product was obtained and sequenced (1,954 bp, accession No. AB901085). The nucleotide sequence encoding PaXynG was found to be 91% identical to that encoding PaXynT over the range −600 to 1,390 of T-34. The deduced amino acid sequence (341 amino acid residues) of PaXynG is 98% identical to that of PaXynT.

### Purification and characterization of PaXynG

**Figure 2:**
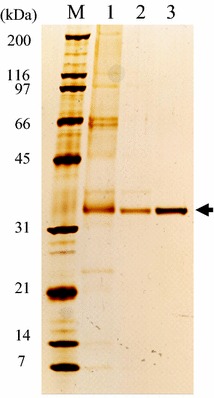
SDS-PAGE of PaXynG of *P. antarctica* GB-4(0). *Lane M* molecular standard, *lane 1* culture medium, *lane 2* TSK-Gel G3000SW_XL_-purified PaXynG, *lane 3* Phenyl Superose HR 5/5-purified PaXynG.

The PaXynG was purified by gel-chromatography and hydrophobic interaction-chromatography as described in Table 3. Purification to homogeneity was confirmed by observation of a single 33-kDa band on a silver stained SDS-PAGE gel (Figure 2). Its specific activity was about 52 U/mg.

**Table 3:** Purification of *P. antarctica* GB-4(0) xylanase (PaXynG)

	Total protein (mg)	Total activity (U)	Specific activity (U/mg)	Yield (%)	Purification (fold)
Crude culture	90.0	1,287.2	14.3	100	1
G3000SW_XL_	18.0	924.8	51.4	71.8	3.6
Phenyl Superose HR 5/5	5.9	306.0	52.3	23.8	3.7

Its optimal pH was 5.2, and the optimal temperature was 50°C (Table 4). It was stable over a wide pH range (3.0–8.0), retaining 97% of their original activities after 1 h incubation (data not shown). The temperature at which the enzyme lost half of their activity (a measure of thermostability) was 57°C. It had no *β*-xylosidase activity. The same results were obtained with purified PaXynT (Table 4).

**Table 4:** Biochemical characterization of PaXyns of *P. antarctica* T-34 and GB-4(0)

Biochemical characters	PaXynT of T-34	PaXynG of GB-4(0)
Optimal pH	5.2	5.2
Optimal temperature	50°C	50°C
pH stability	3.0–8.0	3.0–8.0
Thermostability	57°C	57°C
β-Xylosidaze activity	ND	ND

*ND* not detected.

**Figure 3:**
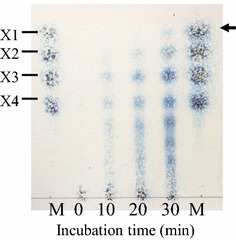
TLC analysis of products resulting from the hydrolysis of beechwood xylan by purified PaXynG for the indicated incubation times. Sugar standards (M) correspond to xylose (X1), xylobiose (X2), and xylotriose (X3). The spot indicated by the *arrow* is xylose (X1).

A TLC analysis of products revealed that purified PaXynG hydrolyzed beechwood xylan to xylose (X1), xylobiose (X2), xylotriose (X3), xylotetraose (X4), and other xylooligosaccharides (Figure 3). The released xylose was confirmed by detection with d-xylose assay kit (data not shown).

### Production of highly concentrated PaXynG by xylose-fed-batch cultivation using a jar-fermentor

**Figure 4:**
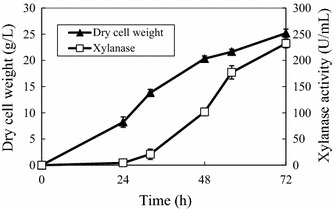
PaXynG production by *P. antarctica* GB-4(0) with xylose feeding using a jar fermentor. Cell growth (*closed triangles*) and PaXynG production (*open squares*) with 2% xylose. Xylose feeding began after 24 h. Each result is the average of two different experiments. *Error bars* show standard deviations.

Because *P. antarctica* GB-4(0) produced larger amounts of PaXyn than T-34 in flask cultivation, GB-4(0) was used in jar fermentor cultivation. After the initial low concentration of xylose (2%) was consumed (24 h), xylose was fed into the PaXyn production medium. This resulted in a DO value around 50% of saturation (data not shown); and 232.4 U/ml (corresponding to 4.5 g/l) of PaXynG was obtained after 72 h (Figure 4).

### Simultaneous production of PaXynG from LBDW during its treatment using a jar-fermentor

**Figure 5:**
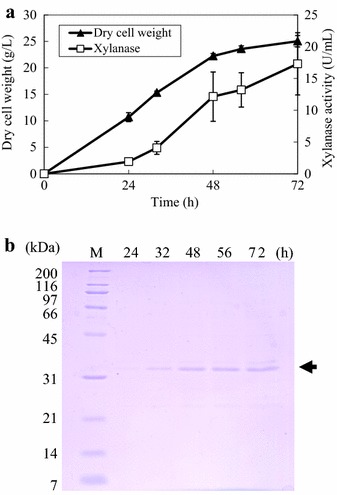
LBDW-fed cultivation of *P. antarctica* GB-4(0) using a jar fermentor. **a** Cell growth (*closed triangles*) and PaXynG production (*open squares*) with 4-times-diluted LBDW. LBDW feeding began after 24 h. Each result is the average of two different experiments. *Error bars* show standard deviations. **b** SDS-PAGE of supernatants (5 μl) periodically sampled from the jar fermentor cultivation. The *arrow* at 33 kDa indicates PaXynG bands.

In a preliminary examination, we found that *P. antarctica* GB-4(0) and T-34 could not grow on LBDW unless it was diluted 4–5 times with water (data not shown). Thus, LBDW-fed-batch cultivation was initially carried out with 4-times-diluted LBDW. After the initial carbon sources, that could be utilized by *P. antarctica* GB-4(0), was consumed (24 h), non-diluted LBDW was fed into the culture using a peristaltic pump. This resulted in a DO value around 60% of saturation (data not shown); 17.3 U/ml (0.3 g/l) of PaXynG was obtained after 72 h (Figure 5a). PaXynG was detected as a 33-kDa band on a CBB stained SDS-PAGE gel (Figure 5b).

In terms of wastewater treatment, over 99% of the xylose content of the LBDW was consumed after 72-h cultivation of *P. antarctica* GB-4(0). The resulting effluent DOC and DTP were 12,000 and 48 mg/l, respectively. The total amount of influent DOC ((26,000 mg + 104,000 mg)/4 l) and DTP ((300 mg + 1,200 mg)/4 l) were calculated as 32,500 and 375 mg/l, respectively. Thus, considering these values, the removal ratios were calculated to be 63 and 87%, respectively. Day removal ratio of DOC (6.8 kg/m^3^/day) was much higher compared to that removed by activated sludge (0.5–1.0 kg/m^3^/day) (Watanabe et al. 2013a).

## Discussion

In this study, we attempted to produce useful materials from LBDW by cultivating *P. antarctica* during treatment of this wastewater. We first identified the highly-secreted 33-kDa unknown proteins in the culture of *P.antarctica* GB-4(0) and T-34 cultivated with xylose to be novel xylanases (PaXyns). Next, we determined the genomic sequence for PaXyn of *P.antarctica* T-34 (PaXynT) using the annotated whole genome sequence (Morita et al. 2013). *P. antarctica* GB-4(0) produced higher PaXyn than *P.antarctica* T-34 (Figure 1). Using the sequence encoding PaXynT, we successfully identified the genomic sequence for PaXyn of *P. antarctica* GB-4(0) (PaXynG). Xylanases are classified into two substantial groups, GH 10 and 11 family, on the basis of their structures (Kimura et al. 2010). The sequence similarity of these PaXyns indicated that they belong to GH10 family xylanases which exhibit higher affinity for shorter liner *β*-1,4-xylooligosaccharides than GH11 family xylanases (Biely et al. 1997).

The optimal pH, optimal temperature, pH stability, and thermostability of the both PaXyns are almost the same (Table 4). They produced xylose (X1), xylobiose (X2), xylotriose (X3), and other xylooligosaccharides from xylan (Figure 3) and did not have any *β*-xylosidase activity (Table 4). These data indicate that they are endoxylanases (1,4-β-d-xylan xylanohydrolases (EC 3.2.1.8)). Since these PaXyns did not have any *β*-xylosidase activity, they could not hydrolyze xylobiose to xylose. Thus, the detected xylose might have resulted from the hydrolysis of xylotriose and other xylooligosaccharides by PaXyns. With this result, it is possible that just like the other endoxylanases (Biely 1985), PaXyns could be employed in saccharizing lignocellulosic biomass materials for the production of useful products.

The production of PaXyns was strongly induced by xylose; a yield of 4.5 g/l of PaXynG was produced by *P. antarctica* GB-4(0) in 72 h (0.0625 g/l/h) with xylose fed-batch cultivation using a jar fermentor (Figure 4). This yield is comparable with 8.1 g/l of recombinant xylanase (PtxynA) of *Paecilomyces thermorphila* produced by a methylotrophic yeast *Pichia pastoris* in 228 h (0.0355 g/l/h) with methanol fed-batch cultivation using a jar fermentor (Fan et al. 2012). The results show that *P. antarctica* is an attractive producer of native xylanase. After centrifugation, about 3 l of supernatant was obtained from 3.5 l of jar culture. The rate of xylanase production (produced PaXynG, 4.5 g/l × 3 l = 13.5 g)/(consumed xylose, 60 g + 200 g = 260 g) by this strain was calculated to be 0.052 g/g.

The hydrolysate of lignocelluloses contains not only fermentable sugars but also toxic compounds that inhibit cell growth, such as furans and organic acids (Okuda et al. 2008). Since these compounds are also generated during fermentation and distillation processes, they are expected to be present in LBDW. It is probably the reason why *P. antarctica* GB-4(0) could not grow in non-diluted LBDW. However, with LBDW fed-batch cultivation, about 0.3 g/l of PaXynG was produced by *P. antarctica* GB-4(0) in 72 h. After centrifugation, about 3.5 l of supernatant was obtained from 4.0 l of jar culture. This is equivalent to the xylanase production rate of about 0.006 g/g (produced PaXynG, 0.3 g/l × 3.5 l = 1.05 g/consumed xylose of LBDW, 34.5 g + 134 g = 168.5 g).

PaXynG production by *P. antarctica* GB-4(0) from LBDW, resulted in the removal of 63% of DOC and 87% of DTP. Because ammonia solution was added for pH control and nitrogen source, DTN removal ratio could not be accurately estimated. However, since the initial DTN was low, the residual DTN is expected to consist mainly of PaXynG which could be easily recovered by ultrafiltration. In the case of *shochu* distillery wastewater, we previously confirmed that the yeast-treated wastewater was efficiently treated by a combination of nitrification/denitrification cycle treatment and activated sludge process (Watanabe et al. 2009, 2013c). In a laboratory-scale demonstration, 50 cycles (25 days) removed 98.9% of DOC, 95.7 of DTN, and 94.1% of DTP from barley *shochu* distillery wastewater (Watanabe et al. 2013c). Thus, it can be expected that the remaining DOC, DTN, and DTP of *P. antarctica* GB-4(0)-treated LBDW could also be easily removed by additional conventional wastewater treatment methods. These results indicated that xylanase production by *P. antarctica* GB-4(0) from LBDW could also contribute to the cutting of LBDW treatment cost.

## Abbreviations

COD: chemical oxygen demand
DOC: dissolved organic carbon
DO: dissolved oxygen
DTN: dissolved total nitrogen
DTP: dissolved total phosphorus
FMM: fungal minimum medium
LBDW: lignocellulosic bioethanol distillery wastewater
PaXynG: xylanase of *P. antarctica* GB-4(0)
PaXynT: xylanase of *P. antarctica* T-34
SDS-PAGE: Sodium dodecyl sulfate-polyacrylamide gel electrophoresis
TLC: thin-layer chromatography
